# Machine learning algorithm for estimating karst rocky desertification in a peak-cluster depression basin in southwest Guangxi, China

**DOI:** 10.1038/s41598-022-21684-5

**Published:** 2022-11-09

**Authors:** Yali Zhang, Yichao Tian, Ying Li, Donghua Wang, Jin Tao, Yongwei Yang, Junliang Lin, Qiang Zhang, Luhua Wu

**Affiliations:** 1 School of Resources and Environment, Beibu Gulf University, Qinzhou, 535011 China; 2 Key Laboratory of Marine Geographic Information Resources Development and Utilization in the Beibu Gulf, Beibu Gulf University, Qinzhou, 535011 China; 3 College of International Studies, Beibu Gulf University, Qinzhou, 535011 China; 4 College of Environmental Science and Engineering, Guilin University of Technology, Guilin, 541004 China; 5 School of Economics and Management, Tongren University, Tongren, 554300 China

**Keywords:** Ecology, Environmental sciences, Natural hazards

## Abstract

Karst rocky desertification (KRD) has become one of the most serious ecological and environmental problems in karst areas. At present, mapping KRD with a high accuracy and on a large scale is still a difficult problem in the control of KRD. In this study, a random forest (RF) based on maximum information coefficient and correlation coefficient feature selection is proposed to predict KRD. Nine predictors stood out as feature factors to estimate KRD. Rock exposure was the most important predictor, followed by fractional vegetation cover for the prediction of KRD processes. The kappa and classification accuracy indexes were to evaluate the performance of the model. We recorded overall accuracy rate and kappa index values of 94.7% and 0.92 for the testing datasets respectively. The RF model was then used to predict the KRD in 2001, 2011, 2016, and 2020, and it was found that the KRD in the study area has exhibited a positive trend of improvement. Therefore, the use of multisource remote sensing data combined with the RF model can obtain better prediction results of KRD, thereby providing a new idea for large-scale estimation of the KRD in peak-cluster depression.

## Introduction

Karst rocky desertification, similar to desertification, is a landscape characterized by a large area of exposed bedrock due to vegetation destruction and soil erosion in karst areas^1,2^. It is not only a process of land degradation but also the result of land degradation. Typically, karst rocky desertification areas are characterized by thin surface soil, weak land productivity, and a poor anti-disturbance ability. Its ecological and environmental problems have been successively identified ^3^, including an extremely fragile ecological environment, loss of biodiversity^4^, and ecosystem degradation^5,6^. The ecological environmental security problems caused by karst rocky desertification have seriously affected people's living environment and sustainable development^7^, and thus, KRD has drawn intensive interest in the field of global environmental change^8^.

Remote sensing technology has been widely used in military, agricultural, medical, and geographical mapping research due to its advantages of fast acquisition, high resolution, low cost, and good security. With the advancement of remote sensing techniques, karst rocky desertification assessment based on remote sensing data has also been rapidly developed^9^. This is exemplified in the work undertaken by Liu et al.^10^, which involved using a multispectral remote sensing Landsat 8 image to calculate the brightness temperature and determining the degree of karst rocky desertification by setting the brightness temperature threshold in Pingguo County, Guangxi. Another study was conducted by Zhang et al.^11^, in which based on a hyperspectral Hyperion image, the abundances of vegetation and exposed rock were extracted to monitor and evaluate the karst rocky desertification using the retrieved annual vegetation coverage from medium-resolution Moderate Resolution Imaging Spectroradiometer (MODIS) data. In a similar case, Zhang et al.^12^ assessed karst rocky desertification (KRD) in southwestern China. The information on karst rocky desertification was extracted using high spatial resolution GF-1 wide field of view (WFV) satellite data for the Nandong underground river basin. Overall, the remote sensing data used to monitor the degree of karst rocky desertification have transitioned from multispectral^13^ to hyperspectral images^14,15^, and low resolution data have been replaced by high resolution data^16,17^.

Based on remote sensing data, considerable research has been conducted on karst rocky desertification monitoring techniques and methods. Traditionally, satellite imagery for karst rocky desertification mapping has relied on visual interpretation or human–computer interactive interpretation ^18^. This is evident in the case of Huang and Cai^19^, in which the human–computer interaction interpretation method was used to interpret remote sensing images acquired in 1974, 1993, and 2001, and then, the spatial pattern and changes in the karst rocky desertification in the middle of Guizhou Province were analyzed. Similarly, based on Thematic Mapper (TM) remote sensing data, Wang et al.^20^ mapped the karst rocky desertification in northern Guangdong through visual interpretation, and the accuracy of their karst rocky desertification map interpretation reached 93.6%. Although human visual interpretation can more accurately classify karst rocky desertification from remote sensing images, it demands considerable professional knowledge and is always time-consuming, which strongly hinders its efficiency. Therefore, it can only be used for the assessment of the degree of karst rocky desertification on a small scale, making it difficult to conduct such research over a large area^4^. To overcome these limitations, scholars have used unsupervised classification, spectral hybrid analysis, and machine learning algorithms to extract karst rocky desertification information. For example, Li and Wu^13^ used the decision tree and fuzzy maximum likelihood methods to classify KRD. When the vegetation fraction, bedrock exposure, and slope factor were added to the classifier, the classification accuracy improved from 84.23 to 91.71%. Chen et al.^21^ utilized the Classification And Regression Tree (CART) method, which increased the unsupervised classification, and normalized difference vegetation index (NDVI) data participation decision classification to classify the KRD, which effectively avoided the problem of artificial subjectivity. After this, based on Advanced Land Observing Satellite (ALOS) imagery, Qi et al.^22^ assessed the feasibility of using the dimidiate pixel model (DPM) and spectral mixture analysis (SMA) approaches for KRD monitoring. Alternatively, a combination of spectral analysis and the vegetation index can be used to extract karst rocky desertification information with a high accuracy, but this method has difficulty identifying the degree of karst rocky desertification in similar shaded areas^23^. Supervised classification and spectral hybrid analysis can speed up the annotation process. However, high-quality expert-annotated samples are still a prerequisite for achieving accurate results using intelligent approaches. Unsupervised classification, to a certain extent, avoids the subjectivity of the artificial selection of samples, but has difficulty distinguishing between types of ground objects with small spectral characteristic differences. In the background of the big data era, machine learning methods such as support vector machines, random forest models, and neural networks have been extensively used in the fields of hydrology, meteorology, and ecology, and they have also provided a new direction in the extraction of karst rocky desertification information^24^. Machine learning combined with the factors influencing karst rocky desertification can not only overcome human subjectivity, but also efficiently identify the degree of karst rocky desertification in large areas^4^.

A recent case study reported by Zhang et al.^25^ argues that the optimal factor influencing karst rocky desertification is of great significance to the evaluation of the degree of karst rocky desertification in karst areas. The vegetation coverage, rock exposure rate, and slope are usually used as grading factors for the degree of karst rocky desertification^26,27^. However, the factors influencing karst rocky desertification are complex and diverse. In support of this, Huang et al.^8^ utilized artificial neural networks (ANNs) to identify the importance of different environmental factors to karst rocky desertification. Zhang et al.^28^ used correlation analysis to study the relationships between karst rocky desertification, temperature, and rainfall and pointed out that karst rocky desertification is not sensitive to responses to climate change and there is a certain lag. Bai et al.^29^ explored the influence of lithology on karst rocky desertification using a combination of mathematical modeling and spatial analysis. Zhang et al.^25^ concluded that the karst rocky desertification index (KRDI) is a good indicator of karst desertification, and the higher the KRDI value, the higher the degree of desertification. In addition, the fragile ecological environment and unreasonable human interactions have promoted the aggravation of karst rocky desertification. For example, Li and Xiong^30^ qualitatively analyzed the impact of human activities on the degree of karst rocky desertification and pointed out that traditional agricultural activities have less impact on karst rocky desertification, while sudden short-term destructive economic activities are the humanistic motivations for initiating large-scale karst rocky desertification. Yao et al.^31^ studied the relationships between the degree of karst rocky desertification and the gross domestic product (GDP) and population density using superposition analysis and concluded that areas with higher population densities and higher GDPs also had higher degrees of karst rocky desertification. Shi et al.^32^ used night light remote sensing data to verify the impact of human activities on karst rocky desertification, and their study showed that the total night light (TL) associated with severe karst rocky desertification was concentrated in Guizhou and Yunnan. In addition, a large and growing body of literature has investigated the temporal and spatial distributions and change characteristics of karst rocky desertification, and these studies have achieved some results^33^. However, the research areas have been concentrated in Guizhou and Yunnan, and the research scale has usually been the county scale^34,35^. Few studies have been conducted on the karst rocky desertification in peak-cluster depression basins^36^.

The karst peak-cluster depression basin in southwest Guangxi is a typical area where tropical karst and non-karst landforms intersect in the world, and it is also one of the hotspots of global biodiversity and ecosystem services. In addition, strong climate change, geological movements, and unreasonable human activities have caused karst rocky desertification to become the most serious environmental problem in this area, threatening the ecological security and economic and social development in the karst region in the peak-cluster depression basin in southwest Guangxi.

In response to these issues, in this study, a typical karst peak-cluster depression watershed in southwest Guangxi was selected as the study area. The main objectives of this study were as follows: (1) to analyze the relevant factors that may affect the development and evolution of karst rocky desertification; (2) to identify the optimal karst rocky desertification characteristic factors; and (3) to invert the spatial and temporal patterns of karst rocky desertification from 2001 to 2020. The results of this study not only provide ideas for karst rocky desertification monitoring in peak-cluster depressions but also provide a data reference for government decision-makers and environmental managers to make macroscopic decisions.

## Results

### Feature factors

The R and MIC scores were used as measurements of linear and nonlinear correlations, and the results shown in Table 1.

**Table 1:** MIC and correlation coefficients on the factors influencing of KRD.

	RE	FVC	S	LAI	FPAR	ET	P	LST	DEM	SP	ST	POP	L	DI
MIC	0.89	0.58	0.36	0.54	0.62	0.54	0.45	0.29	0.27	0.15	0.05	0.23	0.05	0.33
R	0.87	− 0.77	0.48	− 0.69	− 0.76	− 0.66	− 0.19	0.42	0.41	− 0.08	− 0.05	0.24	0.03	0.19

*RE* rock exposure, *FVC* fractional vegetation cover, *S* slope, *LAI* leaf area index, *FPAR* fraction of photosynthetically active radiation, *ET* evapotranspiration, *LST* land surface temperature, *DEM* elevation, *P* precipitation, *SP* slope aspect, *ST* soil type, *POP* population, *L* lithology, *DI* drought index.

**Figure 1:**
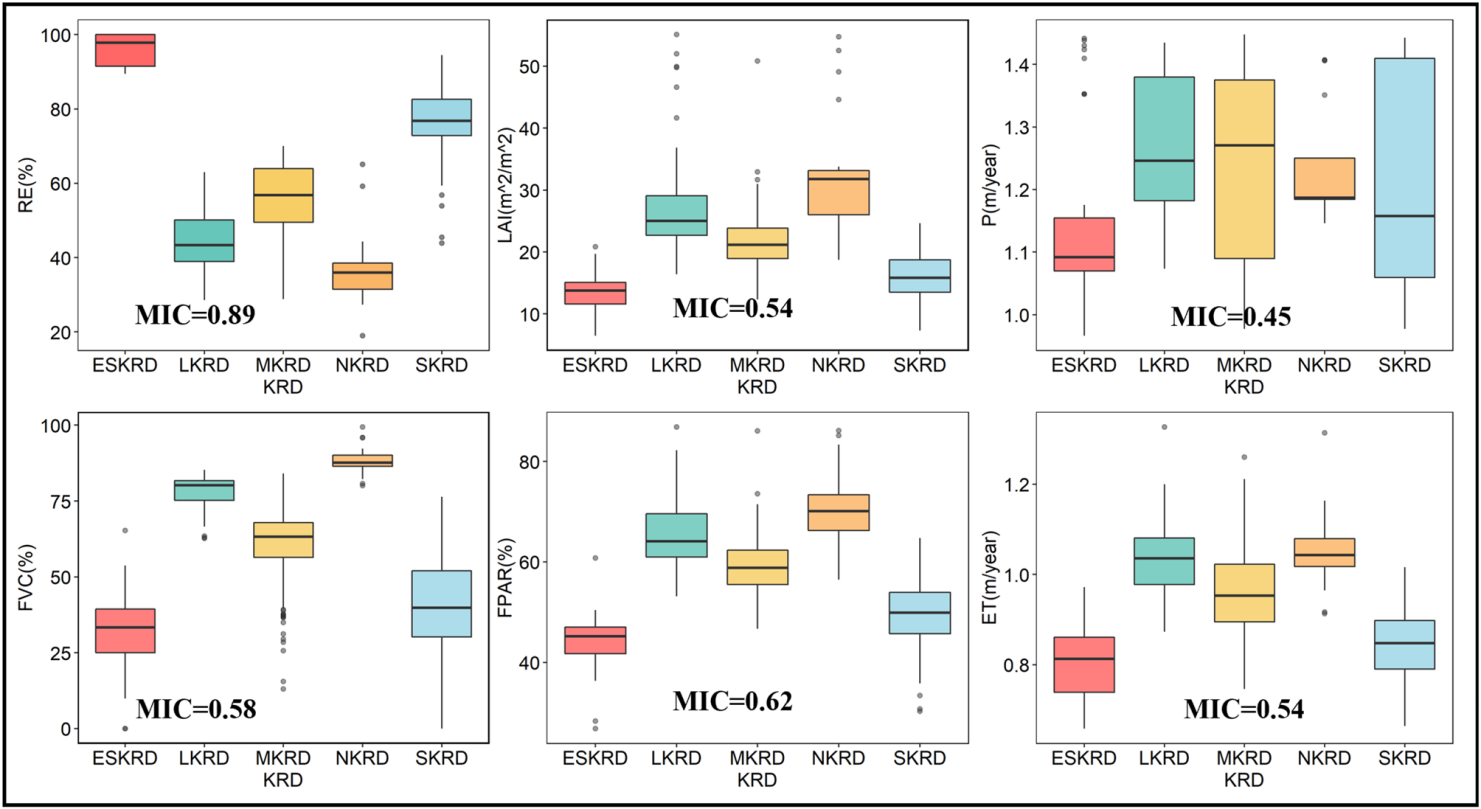
Feature factors of KRD in 2020 selected based on MIC. Abbreviations: NKRD—No karst rocky desertification. LKRD—Light karst rocky desertification. MKRD—Medium karst rocky desertification. SKRD—Severe karst rocky desertification. ESKRD—Extremely severe rocky desertification.

The MIC, which is a nonlinear variable discovery method, revealed that 6 features of the 14 variables were relevant to the KRD in the peak-cluster depression basin in southwest Guangxi (Fig. 1). As is shown in Fig. 1, these six features were the RE, FVC, LAI, FPAR, ET, and P (MIC > 0.4). Among them, the RE and FVC had the strongest impact on the karst rocky desertification.

**Figure 2:**
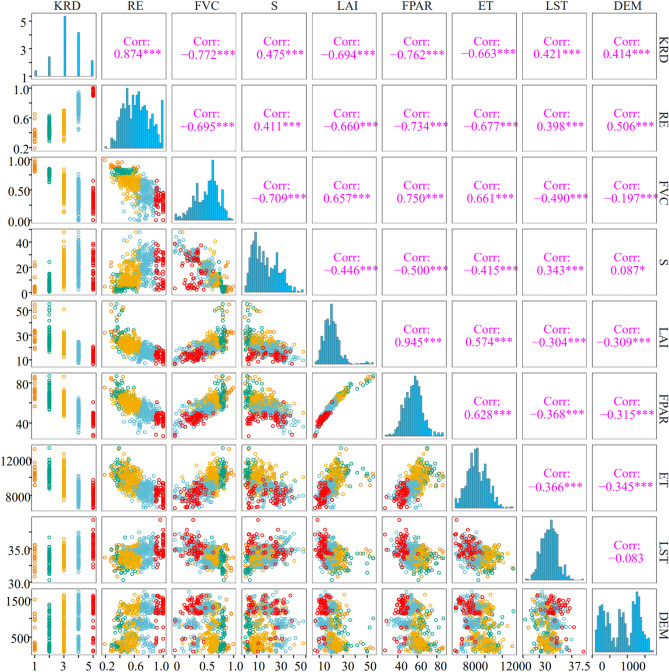
Feature factors of KRD selected based on correlation coefficient. *Significant correlation at the 0.05 level (both sides); **significant correlation at the 0.01 level (both sides); ***significant correlation at the 0.001 level (both sides).

**Figure 3:**
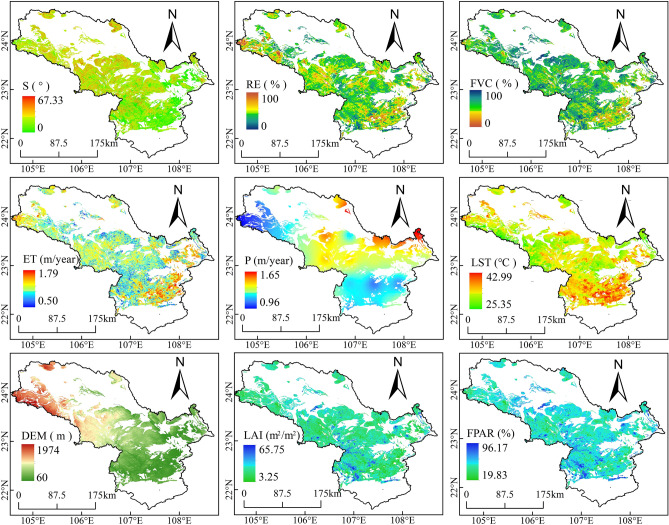
Feature factors of KRD in 2020 selected based on MIC and R in the peak-cluster depression basin in southwest Guangxi, China. Maps were generated using QGIS 3.26.2 (https://www.qgis.org/en/site/).

As can be seen from Table 1, the RE, FVC, S, FPAR, ET, LAI, DEM and LST factors exhibited strong correlations with the degree of karst rocky desertification (Fig. 2), while the correlations between the drought index, lithology, soil type, population density, and slope direction and the degree of karst rocky desertification were low and only revealed the correlation degree between each factor and the karst rocky desertification linearly. Therefore, the RE, FVC, S, LST, P, ET, LAI, DEM and FPAR were selected as the feature factors for inverting the spatial distribution of the karst rocky desertification. Figure 3 shows the selected feature factors via MIC values and correlation coefficients.

### Model accuracy and variable importance

**Figure 4:**
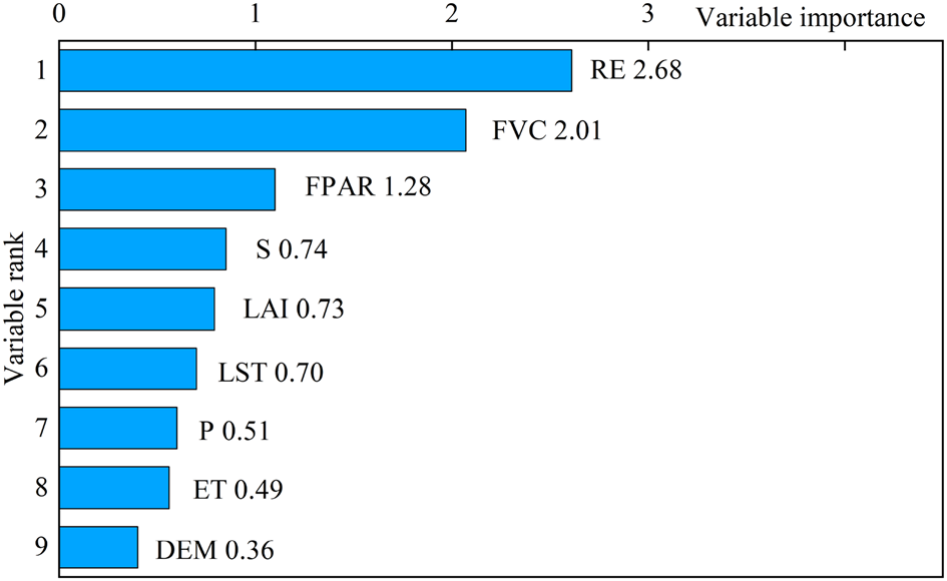
The importance of feature factors of KRD in the peak-cluster depression basin in southwest Guangxi, China.

A summary of the parameters characterizing the accuracy of the RF models is presented in Table 2, which indicates that the overall accuracy rate of the random forest model is 94.7% and the kappa coefficient is 0.92. So, the random forest model is more reliable in mapping the karst rocky desertification. The importance of the input features obtained from the RF model can be used to measure their contributions to the classification accuracy (Fig. 4). Specifically, the variable importance scores are as follows: RE > FVC > FPAR > S > LAI > LST > P > ET > DEM. Notably, as the predominant features, RE and FVC score about twice compared with the following feature FPAR.

**Table 2:** Accuracy of the RF, including the kappa, OA, UA, and PA.

Overall accuracy (OA)	Kappa	User’s accuracy (UA)					Producer’s accuracy (PA)					Run time
		NKRD	LKRD	MKRD	SKRD	ESKRD	NKRD	LKRD	MKRD	SKRD	ESKRD	
94.7%	0.92	100%	88.9%	96.8%	92.7%	92.9%	100%	80%	93.8%	97.4%	100%	4.5 s

### Spatial and temporal distributions of karst rocky desertification

**Figure 5:**
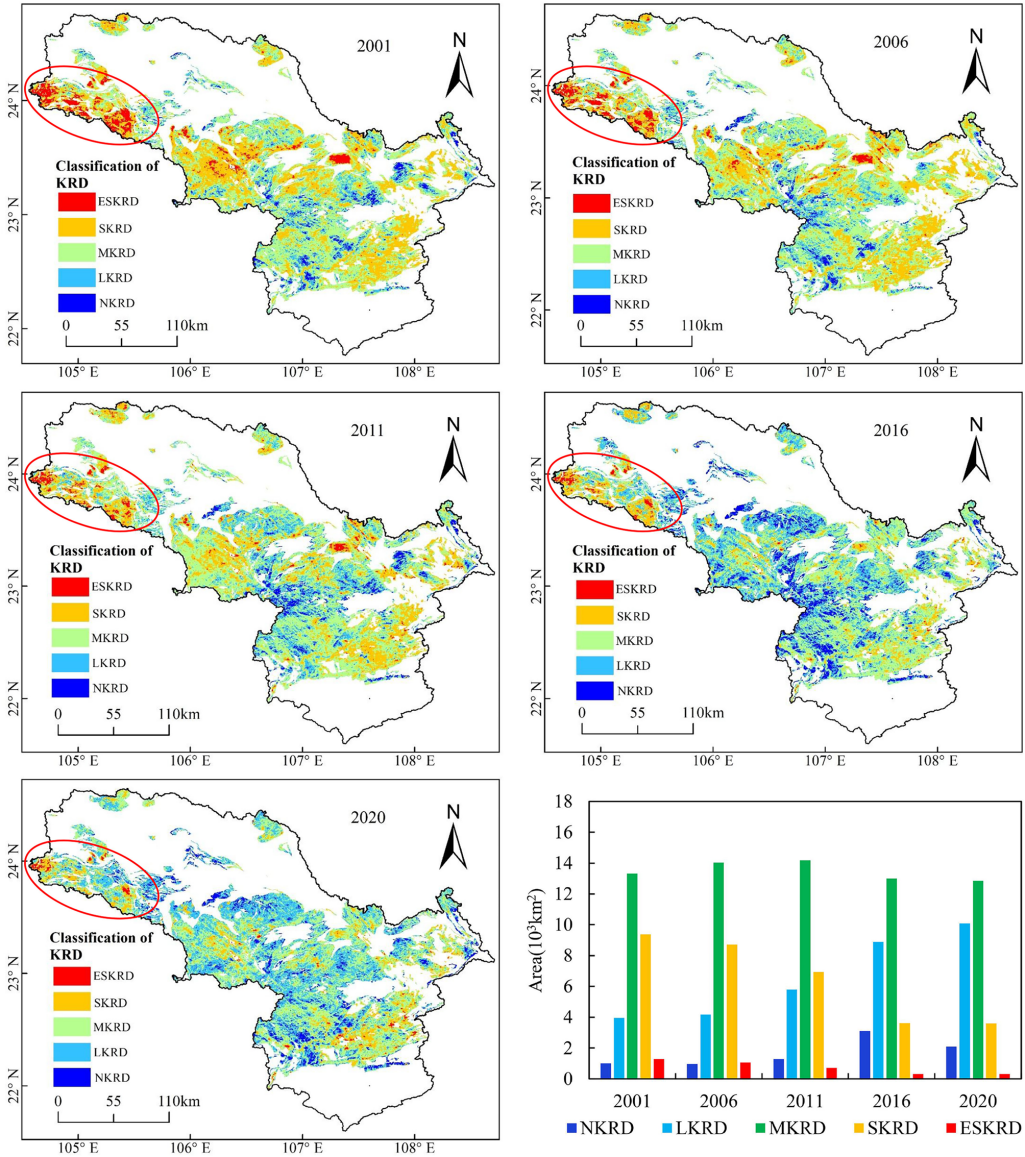
Spatial distribution of the karst rocky desertification**.** Maps were generated using QGIS 3.26.2 (https://www.qgis.org/en/site/).

Figure 5 shows the distribution of the karst rocky desertification in the peak-cluster depression basin in southwest Guangxi. The evolution of the study area was characterized by KRD in space, indicating that the KRD generally improved, and the areas of SKRD and ESKRD continuously decreased. Specifically, in the early part of the study period (from 2001 to 2006), the SKRD and ESKRD accounted for large areas. The macroscopic pattern of the spatial distribution of the karst rocky desertification shows that the upper reaches of the basin were dominated by SKRD and ESKRD, the central region was dominated by MKRD, and the lower region was characterized by LKRD and MKRD. However, from 2011 to 2020, the degree of karst rocky desertification was contained, and the severe and extremely severe karst rocky desertification were scattered in small areas. The macroscopic pattern of the spatial distribution of the karst rocky desertification shows that only a few SKRD and ESKRD areas were distributed in Guangnan County in the upper reaches of the basin.

In general, since 2010, there has been an obvious decrease in the total area of karst rocky desertification in the peak-cluster depression basin in southwest Guangxi, indicating that the karst rocky desertification problem generally exhibits a reverse trend, primarily manifested as a decrease in the level of karst rocky desertification. However, there are still a large number of moderate rocky desertification. In view of this, we suggest: (1) The primary task of karst rocky desertification control should follow the laws of nature, increase protection, reduce human disturbance, and strengthen the protection of potential rocky desertification land. For example, relevant government departments appropriately promote the pace of local urbanization, increase the intensity of ecological migration and rural population transfer, and effectively alleviate the population pressure in karst areas. (2) The ideal policies for the prevention and treatment of the rocky desertification peak-cluster depression basin in southwest Guangxi should properly handle the relationship between economic development and ecological protection so that local residents can gradually change their dependence on their original farming livelihoods. (3) Different methods should be effectively utilised according to the specific karst environments. Ecological restoration work should be carried out scientifically and rationally, particularly in areas with severe rocky desertification.

As can be seen from Table 3, the total area of KRD changed from 27,920 km^2^ to 26,830 km^2^ in the 20 years from 2001 to 2020; and the net area changed to 1090 km^2^ with a reduction rate of 54.5 km^2^ a^−1^. The MKRD, SKRD, and ESKRD areas decreased from 2001 to 2020 in the peak-cluster depression basin in southwest Guangxi. In particular, the proportions of the SKRD and ESKRD areas decreased from 32.39%, and 4.47% to 12.39%, and 1.05%, respectively. Generally, the comprehensive control effect of the karst rocky desertification was remarkable, and the overall karst rocky desertification exhibited a trend of improvement, but LKRD and MKRD were still widely distributed.

**Table 3:** Change in the karst rocky desertification areas of different levels.

Year	LKRD		MKRD		SKRD		ESKRD		Total karst area (10^3^ km^2^)
	Area (10^3^ km^2^)	Proportion (%)	Area (10^3^ km^2^)	Proportion (%)	Area (10^3^ km^2^)	Proportion (%)	Area (10^3^ km^2^)	Proportion (%)	
2001	3.95	13.65	13.31	46.03	9.37	32.39	1.29	4.47	27.92
2006	4.17	14.39	14.04	48.55	8.71	30.11	1.05	3.63	27.96
2011	5.79	20.03	14.19	49.06	6.95	24.02	0.70	2.43	27.63
2016	8.89	30.73	13.00	44.94	3.61	12.48	0.31	1.08	25.81
2020	10.10	34.91	12.85	44.42	3.58	12.39	0.30	1.05	26.83

In the period from 2001 to 2020, the rocky desertification levels in the study area tended to decrease. The reason for the change of karst rocky desertification is mainly due to the rapid development of urbanization as well as social economy, and a large number of rural laborers have shifted from traditional agriculture to other industries, which has slowed down the pressure on land^37^. The reduction in farmland area improved farmland management and increased regional gross industrial product, which together with the continuously rising gross domestic product of the tertiary industry caused a positive rocky desertification development^38^. In addition, the Program of Conversion from Cropland to Forest and Grassland had been applied to restore the vegetation ecosystem since 2005, which has contributed to the gradual development of the ecological and environmental conditions of karst rocky desertification in a benign direction^39^.

## Discussion

The occurrence of karst rocky desertification is a dynamic evolutionary process in time and space, and it is the result of the joint influences of the natural environment and human activities^40–42^. The formation factors of karst rocky desertification are complex and diverse, and an inversion model of karst rocky desertification needs to combine reasonable karst rocky desertification feature factors to produce reliable results. Feature extraction or generation is a critical step in the recognition process since the designated attributes strongly influence the recognition results^43^. Too many of the variables available may introduce noise or may not provide information to identify KRD^44^. When the feature factors used in the machine learning model are insufficient, the model will be under-fitted, which will lead to a certain deviation in the predicted results. However, adopting too many features will increase the search space of the model and the run time of the model, and concurrently, the corresponding model’s construction process will be more complicated^45^. In addition, irrelevant factors will interfere with the model. Therefore, it is necessary to strategically identify the variables related to KRD, which can produce the best effect from the karst rocky desertification prediction model. Related studies have pointed out that the rock exposure rate and vegetation cover contribute the most to karst rocky desertification extraction^17^, which is consistent with the findings of this study.

As is shown in Table 1, the MIC and correlation coefficient between the KRD and RE are 0.87 and 0.89, respectively. In addition, the results of the variable importance assessment provided in Fig. 4 indicate that the RE and FVC were the two most important variables affecting karst rocky desertification in the peak-cluster depression in southwest Guangxi. This was also proved by Xi et al.^46^ and Gu et al.^31^. From these two studies, it is evident that the NDVI had the best correlation with the karst rocky desertification, and the RE had a significant positive correlation with the occurrence intensity of the karst rocky desertification. Specially, based on geographical detector technology, Xi et al. obtained the contribution rates of the RE and FVC to karst rocky desertification, which were 44% and 42%, respectively. Gu et al. measured the importance of various factors to karst rocky desertification using the partial least squares regression model (PLS). However, this study attempted to input only the rock exposure rate and the vegetation coverage into the machine learning model, and the classification effect of the model was not good.

Moreover, data from several studies suggest that the leaf area index, LST, slope, rainfall, and ET can also be used as important factors and indicators for analyzing and evaluating the degree of rock desertification. For example, using Landsat 8 data, Deng et al.^47^ summarized the spatial distribution of the LST in karst areas, revealing that land surface temperature can describe the characteristics of karst rocky desertification in karst areas to a certain extent. Likewise, Li et al.^48^ found that biophysical parameters such as the surface vegetation coverage and leaf area index can better reflect the distribution of karst rocky desertification. Overall, these results demonstrate that the LST and LAI can better reflect the distribution of karst rocky desertification, which is consistent with the results of this study. As is shown in detail in Table 1, the LAI and FPAR also exhibit good correlations with the degree of karst rocky desertification. When all of the factors were incorporated into the machine learning model, the accuracy of the model classification did not improve (kappa coefficient was 0.86), but the run time of the model was longer. Thus, the scientific selection of the feature factors is particularly important when inverting karst rocky desertification. In this study, the feature variables selected using the MIC and correlation coefficient were added to the random forest model, which increased the overall accuracy of the model classification to 94.7% and the kappa coefficient to 0.92.

Traditional karst rocky desertification monitoring methods mainly rely on ground surveys, which require a great deal of time and money. Owing to the terrain limitations, they can only be carried out in areas with low altitude slopes and are not suitable for investigation in peak-cluster depression areas^49^. Although visual interpretation of remote sensing images for monitoring rock desertification is not limited by the topography, it also has disadvantages, including a low interpretation efficiency, easily influenced by human subjectivity, and difficulty guaranteeing the accuracy. In this study, based on machine learning, the spatial distribution of the karst rocky desertification in a karst area was mapped using remote sensing data and auxiliary data. Using a traditional machine learning algorithm has certain advantages, but the selection of the feature vectors and the determination of the model parameters all have a certain impact on the accuracy of the prediction model^50^. Recently, little research has been conducted on mapping karst rocky desertification information based on machine learning algorithms. Pu et al.^17^ compared the accuracy of three algorithms, i.e., the random forest (RF), bagged decision tree (BDT), and extreme random tree (ERT) algorithms, and determined that their overall accuracies (OAs) were 85.21%, 80.85%, and 78.93%, respectively. Xu et al.^4^ used a support vector machine model (SVM) to evaluate the karst rocky desertification areas in Liujiang, Changshun, and Zhenyuan. The overall accuracies in these areas were 85.50%, 84.00%, and 84.86%, respectively; and the kappa coefficients reached 0.8062, 0.7917, and 0.8083, respectively. Obviously, the differences in the research areas and the setting of the model parameters have a certain influence on the accuracy of the prediction model^45^. Some scholars have proposed that among many machine learning algorithms, the RF algorithm has the advantages of simple training, high computational efficiency, and high stability in the changing of parameter values in a classification model^51^. Although RF algorithm was faster to train and more stable, The accuracy of the random forest model depends on the settings of the internal parameters^52^. So, in our study, an iterative backward feature elimination procedure was used to reduce the number of less relevant variables until the internal accuracy (calculated on the basis of the OOB error) no longer varies. Using this approach significantly increases classification accuracy^53^. In addition, another limitation of the random forest model is that the accuracy of the model depends on the quality of the samples. Previous studies have reported that the sizes of the training samples sets were found to influence the performance of the RF classifier^54^. So, in order to reduce misclassification, the sensitivity of RF classification to the sampling design also needs to be considered^53^. According to the results of many experiments, the model has the highest accuracy when the training set is total of 75% and the test set is 25% in this study. In this study, the RF model, which was optimized via iteration, was used to map the KRD in the peak-cluster depression basin in southwest Guangxi based on machine learning. The overall accuracy of identifying the karst rocky desertification was 94.7%, and the kappa coefficient was 0.92. Therefore, this study provides an effective method of KRD monitoring.

Although quantitative analysis of the driving factors of karst rocky desertification development and evolution was conducted in this study, the in-depth relationships and causality between the different influencing factors need to be explored further. Therefore, in future research, it is necessary to comprehensively consider more factors affecting karst rocky desertification to better reveal the development and evolution mechanisms of karst rocky desertification. In addition, hyperspectral images with a high spectral resolution and rich texture features would be very suitable for the study of karst rocky desertification areas, and they have been widely used in other fields. Thus, a machine learning model based on hyperspectral data, combined with optimized algorithms, is a new way to extend remote sensing image information extraction techniques in karst rocky desertification areas.

## Conclusions

Based on RF classifiers and using multisource remote sensing imagery, the spatio-temporal patterns of karst rocky desertification in the peak-cluster depression basin in southwest Guangxi, China, were monitored. The main conclusions are as follows:

In this study, six feature factors were identified using an MIC value of > 0.4 as the selection standard, and the Pearson correlation method was sued to filter the variable set. Concurrently, based on the results of these two filters, nine factors (RE, FVC, SLOPE, LAI, FPAR, ET, P, DEM and LST) were selected as the optimal factors for the inversion of the karst rocky desertification.

According to the nine feature factors, the RF algorithm was optimized via iteration. The optimized RF model was then used to predict the KRD in 2001, 2011, 2016, and 2020. The overall accuracy of the RF model was 94.7%, and the kappa coefficient was 0.92.

In general, the karst rocky desertification in the study area exhibited a positive trend of improvement. Specifically, both the area and the degree of karst rocky desertification decreased. Despite the remarkable effect of the comprehensive management of karst rocky desertification, areas of light and moderate karst rocky desertification are still widely distributed in the study area.

The RF method with the feature selection would be a better method for karst rocky desertification mapping compared with the common method. So, the accuracy of the optimal monitoring scheme in the peak-cluster depression basin in southwest Guangxi, China, could also be investigated for other regions. In addition, our research provides technical support and data sources for the implementation of projects such as returning farmland to forests, soil and water conservation, and rocky desertification prevention and control.

## Materials and methods

### Study area

The peak-cluster depression basin in southwest Guangxi is located in the slope zone from the Guizhou Plateau to Guangxi Basin, in which karst landforms are widely developed. This region is characterized by a fragile ecosystem, large area, wide distribution, and diverse geomorphological types of carbonate rocks. As a typical peak-cluster depression karst area in southwestern China^55^, the geographical position of the study area is 104° 33′–108° 43′ E and 21° 35′–24° 39′ N, covering an estimated area of 61,485.16 km^2^, and its altitude ranges from 500 to 1700 m. In addition, it possesses a typical tropical and subtropical humid and hot monsoon climate, with a mean annual temperature of 13–14 °C and a mean annual precipitation of 900–1600 mm^56^.

**Figure 6:**
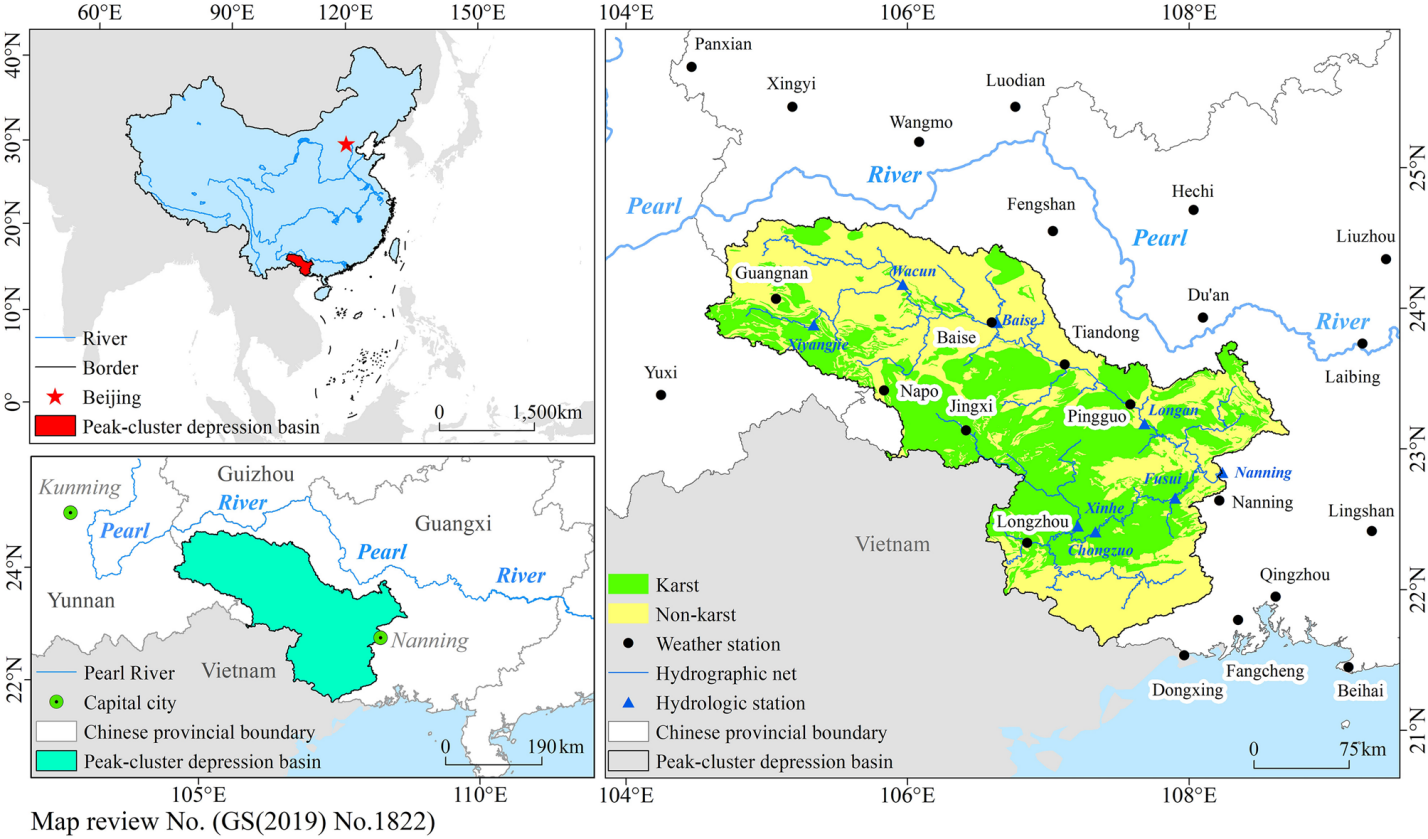
The location of the study area. Maps were generated using QGIS 3.26.2 (https://www.qgis.org/en/site/).

Furthermore, this area is not only an important ecological barrier in the Pearl River Basin, but also an important water conservation area and biodiversity priority protection area in China, as well as one of the areas where ethnic minorities mostly live in the Guangxi Zhuang Autonomous Region^3^. In addition, as a border area, the bilateral political relationships and the situations in neighboring countries have a significant impact on the sustainable development of the ecological environment in the border area. Moreover, the study area is the most convenient sea and land route from China to Vietnam and even Association of Southeast Asian Nations (ASEAN) countries and is an important hub of the “One Belt, One Road” initiative (Fig. 6).

### Data sources and preprocessing

Measured data, as well as a great deal of MODIS remote sensing data and auxiliary data, were employed in this study. Specifically, five MODIS products (MOD09A1, MOD13Q1, MOD11A2, MOD16A3, and MOD15A2) were used (Table 4), and the auxiliary data included elevation, slope, aspect, precipitation, lithology, soil type, drought index, and population data (Table 5).

**Table 4:** Detailed specifications of the remote sensing datasets.

Data Sources	Data type	Variables	Temporal resolution	Spatial resolution	Coverage
MODIS	MOD09A1	Surface reflectance B2, B7	8 days	500 m	Global
	MOD13Q1	NDVI	16 days	250 m	Global
	MOD11A2	LST	Monthly	1 km	Global
	M0D16A3	ET	Yearly	500 m	Global
	MOD15A2	LAI/ FPAR	8 days	500 m	Global

**Table 5:** Relevant parameters and sources of the auxiliary data.

Data	Temporal Resolution	Spatial Resolution	Sources
Soil type (ST)		1 km	https://www.resdc.cn/
DEM		90 m	http://www.gscloud.cn/#page1/2
Slope (S)		90 m	http://www.gscloud.cn/#page1/2
Slope aspect (SP)		90 m	http://www.gscloud.cn/#page1/2
Drought index (DI)	Yearly	0.05	http://climexp.knmi.nl/selectfield_obs2.cgi?id=someone@somewhere
Lithology (L)		Shp	http://www.karstdata.cn/
Population (POP)		1 km	https://www.worldpop.org/geodata/listing?id=64
Precipitation (P)	Monthly	1 km	http://data.cma.cn/

### MODIS data

MODIS data products are widely used in the fields of land use/cover research, natural disaster monitoring and analysis, and marine ecological environment, and they play an important role in ecological environment research and applications at global and regional scales. In this study, the MODIS datasets (tiles h27v06 and h27v062) for 2001, 2006, 2011, 2016, and 2020 were used to calculate the karst rocky desertification. They were all downloaded from the United States Geological Survey (https://earthdata.nasa.gov/). To begin this process, the MODIS Reprojection Tool (MRT) was used for the data extraction, mosaicking, and reprojection. Specifically, the fraction of photosynthetically active radiation (FPAR) and the leaf area index (LAI) products were acquired from MOD15A2. In the same way, the land surface temperature (LST) and evapotranspiration (ET) were retrieved from the MOD11A2. In the follow-up phase, of the seven bands of the MOD09A1 product, only bands 2 and 7 were used to generate the corresponding rock exposure rate (Table 6). After this, the fractional vegetation cover (FVC) was calculated using the NDVI data from MOD13Q1, which provides a 16-day composite with 250 m spatial resolution data, including NDVI products (Table 6). Finally, before further analysis, the above product datasets were uploaded to QGIS3.26.2 (https://www.qgis.org/en/site/), where they were projected to the World Geodetic System (WGS) 1984, Universal Transverse Mercator (UTM) zone 48 N projected coordinate system. They were further resampled to a spatial resolution of 250 m for uniformity. The boundary of the study area was used as a mask for cutting to ensure the same processing extent.

**Table 6:** FVC and RE were **e**xtracted using QGIS3.26.2 (https://www.qgis.org/en/site/).

Factor	Formula	References
RE	$NDRI = \frac{B7 - B2}{{B7 + B2}}$ $RE = \frac{{NDRI - NDRI_{r} }}{{NDRI_{r} - NDRI_{o} }}$	Zhu^57^
FVC	$FVC = \frac{{NDVI - NDVI_{soil} }}{{NDVI_{veg} - NDVI_{soil} }}$	Mishra and Chaudhuri^58^

*NDRI* normalized difference rock index.

### Auxiliary data processing

The sources and details of the auxiliary data are shown in Table 5. These data were processed using the following methods. First, using the QGIS3.26.2 (https://www.qgis.org/en/site/), the soil type, population, precipitation, digital elevation model (DEM), slope, and slope direction data were resampled to the same spatial resolution as the MODIS product data using a nearest neighbor algorithm and by replicating the pixels. Second, the drought index datasets, which were derived from the product data of the Climate Research Unit self-calibrated Palmer Drought Severity Index (CRUscPDSI) with a 1 km spatial resolution, were converted to a point layer. Then, the datasets were interpolated to raster files using the Kriging technique to be consistent with the spatial resolution of the other data.

In particular, the vector data of the lithology data were converted to a raster layer with a 250 m × 250 m spatial resolution for this study. In addition, for uniformity, all of the auxiliary data were further transformed to the World Geodetic System (WGS) 1984, Universal Transverse Mercator (UTM) zone 48 N projected coordinate system. Finally, the auxiliary data were extracted using the study area boundary as a mask to generate the rocky desertification impact factor data.

### Karst rocky desertification survey data

**Figure 7:**
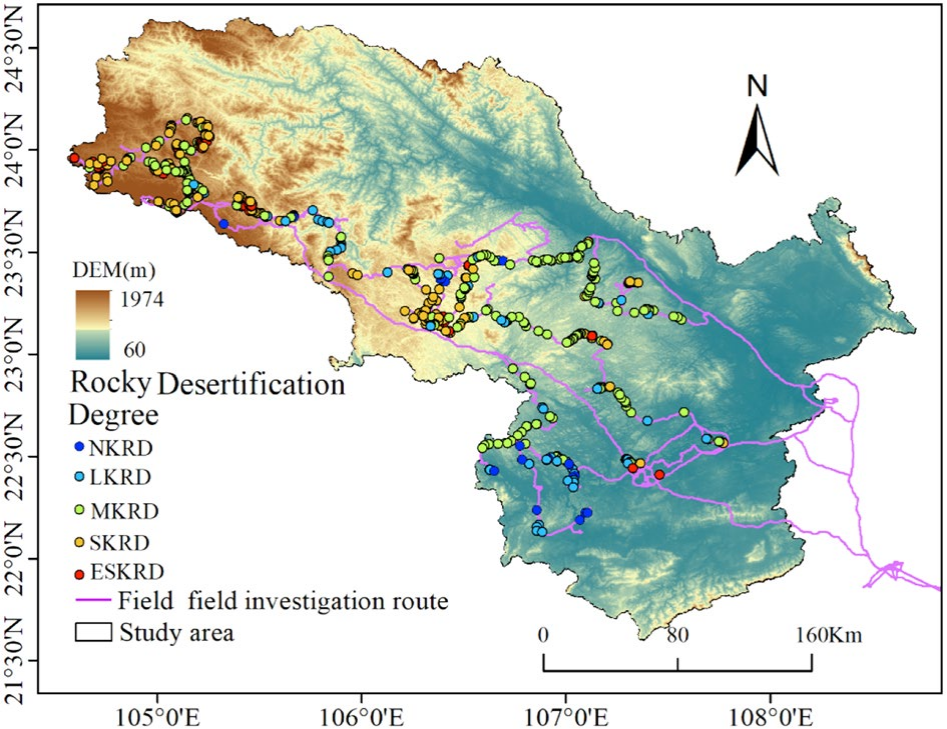
Field data survey map of karst rocky desertification. Map was generated using QGIS 3.26.2 (https://www.qgis.org/en/site/).

The field data were collected during the spring and summer of 2020 (March–August). A total of 527 sampling plots, 30 × 30 m each^17^, were established. They were located randomly along the road so that they would be easy to reach. Within each plot, the longitude, latitude, and elevation of the sample centroids were recorded using a high accuracy global navigation satellite system (GNSS). Then, the area of bare rock and vegetation coverage were measured using a high-precision handheld global positioning system (GPS) measuring instrument. In addition, the vegetation type, landscape type, and surrounding environment were also recorded according to visual observations. Ultimately, the indexes of the vegetation coverage and the rock exposure rate were calculated to determine the classification of the karst rocky desertification (Fig. 7).

Based on the vegetation coverage, rock exposure rate, and rock distribution obtained from the survey, based on previous studies^59^, and combined with the landforms in the study area, the karst areas in this study were classified into five types: non-rocky desertification, light karst rocky desertification, moderate karst rocky desertification, severe karst rocky desertification, and extreme karst rocky desertification. The classification standard is shown in Table 7.

**Table 7:** Field classification standard for karst rocky desertification.

Classification of KRD	Exposure rate of bedrock (%)	Vegetation and soil coverage (%)	Distribution character of the exposed rock
NKRD	< 30	70	None
LKRD	30–50	50–70	Point
MKRD	50–70	30–50	Point, line
SKRD	70–90	20–30	Line, patch
ESKRD	> 90	20	Patch

### Feature selection method

The maximal information coefficient (MIC), which was introduced by Reshef et al. in 2011^60^, is a powerful approach for detecting various relationships between variables, and it was developed on based on mutual information. The larger the MIC value between the two variables is, the stronger the correlation is, and vice versa. And, the Pearson's correlation coefficient, a statistical method that captures the dependence of two variable correlations, is frequently used for the decorrelation of variables and for feature extraction^61^.

### Random forest classifier

The random forest (RF) classifier is a non-parametric ensemble classification method based on a large number of regression trees, especially with two important parameters as the number of decision trees and the number of split nodes^62^. The disadvantage of RF was that the split rules for classification are unknown^63^. However, because of its high stability and its ability to perform efficient processing of large-scale data^64^, the random forest classifier is a more practical integrated learning method, and it can effectively reduce the error of a single classifier and improve the classification accuracy using multiple classifiers for voting classification. Random forest algorithms have randomness in sample and feature selection, which makes it difficult for random forest to fall into overfitting and gives it a good antinoise ability^65,66^. In this study, a bagging integrated random forest classification algorithm was used to predict the degree of karst rocky desertification.

The specific steps were as follows.

First, the degree of karst rocky desertification was defined as the dependent variable, and the vegetation coverage, the rock exposure, and other factors were used as explanatory variables to select the optimal number of leaf nodes by setting RFLeaf = 5, 10, 20, …, 500.

Second, ① the entire dataset was randomly split into calibration (70%) and validation (30%) datasets to model the KRD. The calibration dataset was used to train the models with all of the relevant variables identified by the MIC. The independent validation sets were used to evaluate the predictive performance of the RF model. ② The kappa and classification accuracy indexes were calculated for the validation datasets. ③ If the kappa coefficient was greater than 0.95, the trained model was saved. Otherwise, steps ① and ② were repeated.

Finally, based on the karst rocky desertification characteristic data, the trained RF model was employed to monitor the karst rocky desertification dynamics during1990–2020 in the peak-cluster depression basin in southwest Guangxi.

### Accuracy assessment

The stability and reliability of the model algorithm are the basis of the subsequent research, so it is very important to measure the accuracy of the model. In this study, to test the effectiveness of the RF algorithm, performance measurement metrics, including the overall accuracy, users’ accuracy, producers’ accuracy, and kappa coefficient, were adopted.

### Technical route and workflow

**Figure 8:**
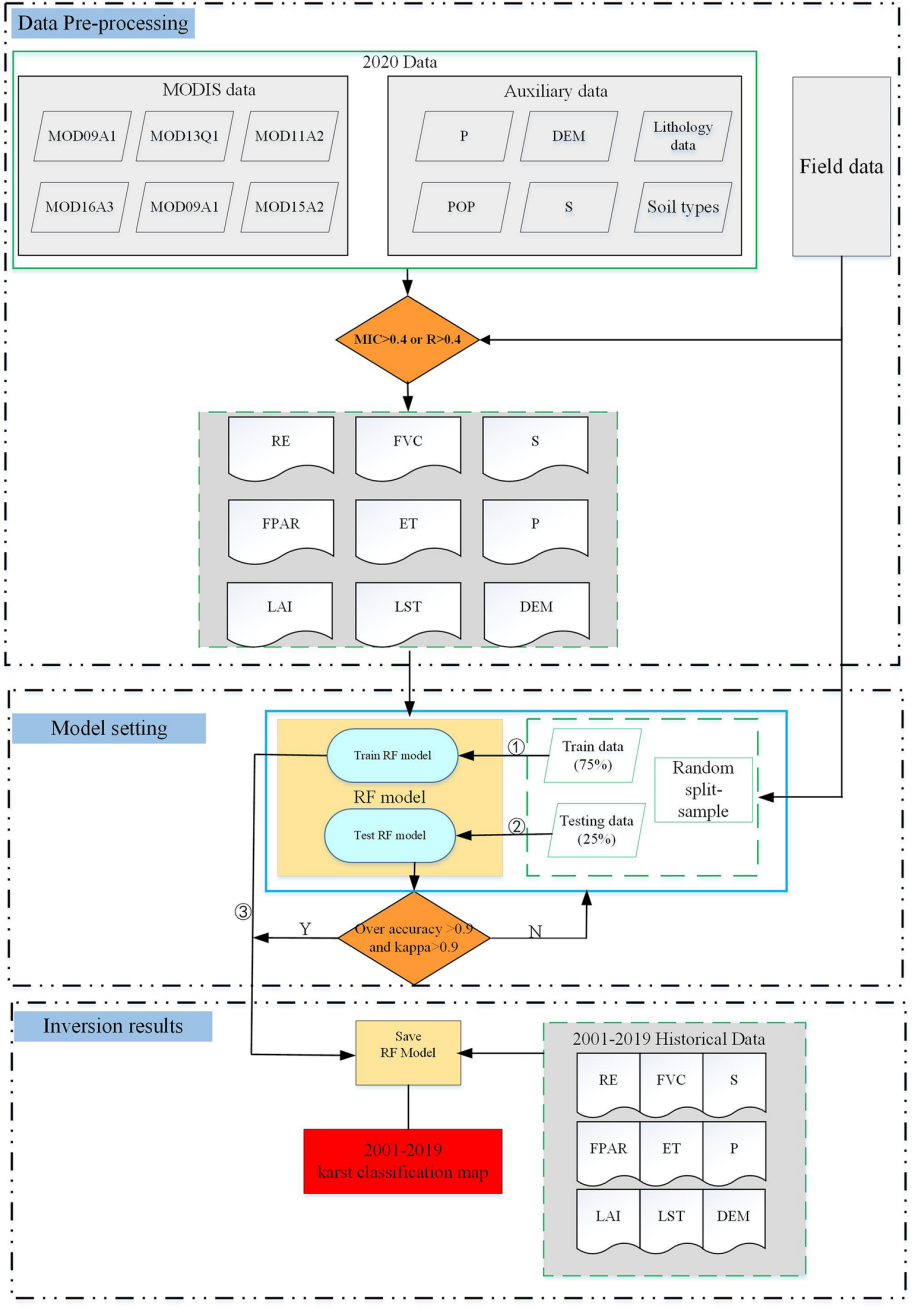
Flow chart of karst rocky desertification mapping in peak cluster depression in Southwest Guangxi, China.

A flowchart of the entire process used in this study is shown in Fig. 8, which can be divided into three parts.

1. Data preprocessing and the extraction of desertification indicators
  Based on the MODIS data and auxiliary data, the karst rocky desertification factor data were extracted, including the vegetation coverage, bedrock exposure rate, surface temperature, leaf area index, photosynthetic utilization efficiency, elevation, slope, slope direction, lithology, soil type, evapotranspiration, population density, and annual precipitation data. The optimum characteristics of the karst rocky desertification factors were selected via the MIC and Pearson's correlation coefficient.
2. RF model setting
  The purpose of this step was to adjust the model parameters. The optimal numbers of trees and leaves for the RF classifier were determined by plotting the Out of Bag (OOB) error versus the number of trees and by determining the threshold number of trees for which the error was stable. The number of trees to be used in the RF classifier was chosen as 250, which is not too computationally expensive but is large enough to stabilize the model error among the ensemble of the decision trees. Similarly, the number of leaves used in the RF classifier was 5, and the OOB error was the smallest.
3. Karst rocky desertification mapping.
  Finally, based on karst rocky desertification feature sets, the trained RF model was used to monitor the karst rocky desertification dynamics during 2001–2020 in the peak-cluster depression basin in southwest Guangxi.

## Data Availability

The data that support the findings of this manuscript are available from the corresponding author, T.Y, upon reasonable request.
